# Characterization of newly isolated oleaginous yeasts - *Cryptococcus podzolicus*, *Trichosporon porosum* and *Pichia segobiensis*

**DOI:** 10.1186/s13568-014-0024-0

**Published:** 2014-03-18

**Authors:** Ines Schulze, Silla Hansen, Steffen Großhans, Thomas Rudszuck, Katrin Ochsenreither, Christoph Syldatk, Anke Neumann

**Affiliations:** 1 Institute of Process Engineering in Life Sciences, Section II: Technical Biology, Karlsruhe Institute of Technology, Engler-Bunte-Ring 1, Karlsruhe, 76131, Germany

**Keywords:** Screening, Oleaginous yeasts, Microbial lipid production, Cryptococcus podzolicus, Trichosporon porosum, Pichia segobiensis

## Abstract

The yeast strains *Cryptococcus podzolicus*, *Trichosporon porosum* and *Pichia segobiensis* were isolated from soil samples and identified as oleaginous yeast strains beneficial for the establishment of microbial production processes for sustainable lipid production suitable for several industrial applications. When cultured in bioreactors with glucose as the sole carbon source *C. podzolicus* yielded 31.8% lipid per dry biomass at 20°C, while *T. porosum* yielded 34.1% at 25°C and *P. segobiensis* 24.6% at 25°C. These amounts correspond to lipid concentrations of 17.97 g/L, 17.02 g/L and 12.7 g/L and volumetric productivities of 0.09 g/Lh, 0.1 g/Lh and 0.07 g/Lh, respectively. During the culture of *C. podzolicus* 30 g/l gluconic acid was detected as by-product in the culture broth and 12 g/L gluconic acid in *T. porosum* culture. The production of gluconic acid was eliminated for both strains when glucose was substituted by xylose as the carbon source. Using xylose lipid yields were 11.1 g/L and 13.9 g/L, corresponding to 26.8% and 33.4% lipid per dry biomass and a volumetric productivity of 0.07 g/Lh and 0.09 g/Lh, for *C. podzolicus* and *T. porosum* respectively. The fatty acid profile analysis showed that oleic acid was the main component (39.6 to 59.4%) in all three strains and could be applicable for biodiesel production. Palmitic acid (18.4 to 21.1%) and linolenic acid (7.5 to 18.7%) are valuable for cosmetic applications. *P. segobiensis* had a considerable amount of palmitoleic acid (16% content) and may be suitable for medical applications.

## Introduction

As world population continues to grow, there is an ever-increasing demand on energy and material resources. Therefore, to ensure long-term sustainability, suitable alternative production methods for oil as feedstock for several industrial applications have to be developed. Biodiesel and bioethanol derived from plant oil for example, have been used since time immemorial, but the disadvantage is the competition with the need to produce feed and food (Ratledge [1993]). Therefore, oleaginous microorganisms represent an alternative production system for sustainable lipid production as they share the special feature to produce more than 20% lipid per dry biomass as carbon storage reserves with similar fatty acid compositions as plant oils.

Lipids are produced by all microorganisms (MOs) usually in the range of 6 to 8% per dry biomass principally as components for the cell membrane. However, oleaginous microorganisms, including yeasts, bacteria, filamentous fungi and microalgae, convert a carbon source when it is available in excess into intracellular triacylglycerols (TAG) as soon as a nitrogen limitation occurs (Ratledge [2002]). These lipids are also called single cell oils (SCO) and are stored as lipid droplets within cells.

Baker’s yeast *Saccharomyces cerevisiae* does not produce any intracellular lipid droplets (Vorapreeda et al. [2012]), but several other yeast strains, however, are known to belong to the oleaginous microorganisms, e.g. *Cryptococcus* sp.*, Yarrowia* sp., *Candida* sp., *Rhodotorula* sp., *Rhodosporidium* sp., *Trichosporon porosum* and *Lipomyces* sp. (Ratledge [1991]). *Cryptococcus curvatus* is one of the most known oleaginous microorganisms able to grow on several carbon sources, e.g. glucose, glycerol or xylose (Meesters et al. [1996]; Zhang et al. [2011]). Advantages of microbial oil production compared to plant oil is the short life cycle of microbes and the possibility of an in vitro production process not influenced by external factors such as venue, season or climate (Thiru et al. [2011]).

The yield and type of lipid is dependent on several factors including the type of microorganism, the culture conditions and the chosen substrates (Li et al. [2008]; Griffiths and Harrison [2009]). Yeast strains produce mainly fatty acids which are similar in composition to those in plant oils containing predominantly saturated or monounsaturated fatty acids with carbon lengths of C16 and C18 (Papanikolaou and Aggelis [2011]). Other microorganisms e.g. microalgae or filamentous fungi are also able to produce significant amounts of poly unsaturated fatty acids (PUFAs) like ω-3 or ω-6 fatty acids, e.g. docosahexaeonic acid (DHA) or eicosapentaenoic acid (EPA) (Ward and Singh [2005]) which are usually extracted from fish oil. This knowledge permits the use of different microorganisms for different industrial applications.

Costs for microbial lipid production include the cost of the raw materials (chosen substrates), costs of the fermentation process (monitoring, control, labour, operating costs) and downstream processing costs. Fermentation costs are almost unchangeable. Substrate costs can be reduced by using low-cost substrates or waste material as carbon and nitrogen source. The greatest challenge for the downstream processing of intracellular lipid is overcoming the high energy costs for the cell disruption.

The establishment of an economic production process entails either attaining higher volumetric output of lipid or producing lipids with high value fatty acids. Therefore suitable microorganisms have to be identified or new strains have to be isolated from the environment which are able to grow on low-cost substrates, e.g. hemicellulosic wastes which include xylose as monomers.

Glucose can be converted by almost every microorganism. Therefore, it is suitable for screening experiments. However, many microorganisms, e.g. *Pichia pastoris* (Lee et al. [1986]) are able to convert xylanes, a constituent of hemicelluloses and polymer of xylose molecules. Hemicellulosic material is accessible in large quantities all over the world (Pan et al. [2009]) and can therefore be classified as low-cost substrate suitable as carbon source for oleaginous microorganisms. Soil samples contain old wood; hence products of decomposition like xylanes might be biotransformed by several soil microbes. Some of those microbes might belong to the group of oleaginous microorganisms showing the ability to convert xylose as carbon source into SCO. New isolates from soil samples may therefore be qualified for the application in biotechnological processes using hemicellulosic feedstock as cost-efficient substrate.

This study aimed to identify new yeast isolates from soil samples for the production of SCO, which can be used as oil substitute for industrial applications in the cosmetic, pharmaceutical, nutritional or energy sectors. Furthermore, xylose as low-cost substrate should be examined and evaluated as carbon source for promising lipid producing isolates.

## Material and methods

### Microorganisms

The oleaginous yeast *Cryptococcus curvatus* (ATCC 20509) was used as a positive control for SCO production. *Saccharomyces cerevisiae* (DSM 11285) as a non oleaginous yeast was taken as the negative control for yeasts not able to produce SCO.

Characterized soil isolates used in this study were deposited at the DSMZ culture collection. CPOH4 *Cryptococcus podzolicus* as DSM 27192, SSOH12 *Pichia segobiensis* as DSM 27193 and TPST6 *Trichosporum porosum* as DSM 27194.

### Soil sample collection

Two samples were taken in summer time from peat bog soil in Kaltenbronn near Bad Wildbad in the black forest of Germany (sample 1: 48.720°N, 8.471°E, 894.4 m above sea level; sample 2: 48.716°N, 8.456°E, 911.8 m above sea level) and one soil sample was taken in summer time from a grassland in Karlsruhe (Baden-Württemberg, Germany, 48.98989°N, 8.40462°E, 116.7 m above sea level). All samples were taken at a depth of 5 cm. The soil samples were stored at −20°C.

### Yeast isolation from soil samples

A fraction of the collected soil sample (10 mg) was resuspended in 1 ml sterile demineralised water. An aliquot (100 μl) of the suspension was plated out on YM agar plates (3 g/L yeast extract, 3 g/L malt extract, 5 g/l peptone, 10 g/L glucose, 20 g/L agar, pH 7) containing antibiotic (10 mg/L ampicillin, 20 mg/L tetracycline). Agar plates were incubated at 20°C until 1 mm diameter colonies became visible. Each colony was picked and viewed under a microscope to determine if it was a yeast. For long-term storage the isolated strains were stored in glycerol stocks (15% w/w) at −80°C.

### Screening for oleaginous microorganisms with Sudan Black B staining

Isolated yeasts were cultivated on YM agar plates for 4 days at 20°C. Replica plates were prepared by transferring the colonies from the original agar plate to a round filter paper (size of agar plate, GE Healthcare Europe GmbH, Freiburg, Germany, Whatman; Ref No 10311610). The filter paper was dried for 15 min at 60°C and subsequently stained for 20 min with 0.08% Sudan Black B in 96%-Ethanol (EtOH) solution under shaking. Afterwards the filter was washed twice for 5 min with 96% EtOH under shaking. Colonies which were stained blue could be potential oleaginous MOs with intracellular TAGs (Evans et al. [1985]).

### Identification of the isolates

Genomic DNA was isolated using the commercial kit “Precellys Bacterial/ Fungal DNA-Kit” (PEQLAB Biotechnologie GmbH, Erlangen, Germany; 12-7511-00). Afterwards polymerase-chain reaction (PCR) fragments were produced applying universal primers ITS1 (5′-TCCGTAGGTGAACCTGCG-3′) (Eurofins MWG GmbH, Ebersberg, Germany) and ITS4 (5′-TCCTCCGCTTATTGATATGC-3′) (Eurofins MWG GmbH, Ebersberg, Germany) (Fujita et al. [2001]). PCR amplification was performed in a total volume of 50 μl. The composition of each PCR reaction was as followed: 5 μl PCR buffer (Dream Taq Green buffer, Thermo Scientific Fermentas, Schwerte, Germany; ♯B71), 5 μl of dNTP mixture (2 mM each) (Thermo Scientific Fermentas, Schwerte, Germany; #R0241), 1 μl ITS1 primer (10 μM), 1 μl ITS4 primer (10 μM) and 0.5 μl Dream Taq DNA polymerase (Thermo Scientific Fermentas, Schwerte, Germany; EP0701) were filled up with PCR water (Carl ROTH GmbH, Karlsruhe, Germany; T143.4). The PCR amplification started with 95°C for initial denaturation, followed by 30 cycles of denaturation at 95°C for 30 s, annealing at 48°C for 30 s, and extension at 72°C for 1 min. The final extension was done at 72°C for 10 min. PCR products were visualized on 1% agarose gel (1× TAE-buffer: 40 mM Tris base, 1 mM EDTA, pH 8 adjusted with acetic acid; 0.1 μg/ml ethidium bromide) after carrying out gel electrophoresis of each PCR amplification product and 6 μl Quick Load 1 kb DNA Ladder (New England Biolabs, Frankfurt/Main, Germany; N0468 S) with 1× TAE buffer at 100 V for 1 h. Distilled water was used as negative control. The amplified DNA (including the 5.8 S rDNA) was sequenced by GATC Biotech Corporation (Konstanz, Germany). Alignments were performed via MEGABLAST with NCBI database (http://www.ncbi.nlm.nih.gov/).

### Cultivation in shake flasks

YM medium (3 g/L yeast extract, 3 g/L malt extract, 5 g/l peptone, pH 7) was supplemented with glucose to an initial concentration of 50 g/L glucose. 50 ml initial culture volume filled in 500 ml conical shake flasks with an initial optical density (OD_600_) of 0.5 were incubated at 130 rpm at 25°C for 120 hours. Every 24 hours 35 g/L glucose was repeatedly added to ensure that the carbon source was in excess.

### Cultivation in bioreactors

For the cultivation in the bioreactor a mineral salt medium was used, formulated on a phosphate buffer at pH 5 (8.99 g/L KH_2_PO_4_ and 0.12 g/L Na_2_HPO_4_*2H_2_O) which was based on the medium used in Meesters et al. ([1996]). The medium constituents were 0.1 g/L sodium citrate*2H_2_O, 0.1 g/L yeast extract, 0.2 g/L MgSO_4_*7H_2_O, 4.72 g/L (NH_4_)_2_SO_4_ (refers to 1 g/L N). Once a day the culture broth was supplemented with 2 ml trace elements solution (4 g/L CaCl_2_*2H_2_O, 0.55 g/L FeSO_4_*7H_2_O, 0.475 g/L citric acid, 0.1 g/L ZnSO_4_*7H_2_O, 0.076 g/L MnSO_4_*H_2_O, 100 μl 18 M H_2_SO_4_) and 2 ml salt solution (20 g/L MgSO_4_*7H_2_O, 10 g/L yeast extract) per 100 ml cultivation medium. Precultures were prepared in conical shake flasks with initial OD_600_ of 0.5 and 120 rpm. Fermentation was performed in a 2.5 L fermentor (Infors HT, Bottmingen, Switzerland; Minifors fermentor) with 1 L initial culture medium, initial OD_600_ of 1, at 600 rpm and with 1 vvm aeration rate without control of dissolved oxygen (pO_2_) for at least 188 h. CPOH4 was grown at 20°C, SSOH12 and TPST6 at 25°C. The control of pH was done automatically by addition of 4 M H_3_PO_4_ and 4 M NaOH in each fermentor, Contraspum A 4050 HAC (Zschimmer und Schwarz GmbH und Co KG, Lahnstein, Germany) was applied as antifoam agent. Initial glucose or xylose concentration was 50 g/L. Each day the carbon source was replenished to a maximum concentration of 90 g/L after determining the actual concentration. A minimum of five samples were taken per day (four samples à 3 ml for the determination of OD_600_, dry biomass (g/L), carbon and nitrogen source and by-products; one sample à 20 ml for lipid analysis (% lipid/dry biomass) via gas chromatography).

### Sample preparation for dry biomass and analysis of supernatant

Dry biomass was analyzed gravimetrically. A 1 ml aliquot of the culture fluid was transferred into a predried and preweighed 1.5 ml reaction tube and centrifuged at 13,000 rpm for 5 min. The supernatant was collected and used for the determination of glucose and NH_4_^+^. The cell pellet was washed with 800 μl saline (0.9% NaCl), dried at 60°C for 24 h and weighed.

### Analysis of NH_4_^+^, glucose, xylose, ethanol, gluconic acid

All components were measured in triplicates with enzymatic test kits. D-Glucose (10716251035), ethanol (10176290035) and gluconic acid (10428191035) were purchased at R-Biopharm AG (Darmstadt Germany). NH_4_^+^ was measured via Spectroquant kit (Merck KGaA, Darmstadt, Germany 1.14752.0001). D-Xylose assay kit was taken for the concentration of xylose (Megazyme, Bray, Ireland; K-XYLOSE).

### HPLC analysis of organic acids

The pure supernatant was taken to measure five different organic acids (gluconic, malic, citric, succinic and fumaric acid) using a standard HPLC device (Agilent 1100 Series, Agilent Technologies Deutschland GmbH, Böblingen, Germany) equipped with a 150 × 4.6 mm HPLC column Synergi™ 4 μm Fusion-RP 80 Å (Phenomenex, Aschaffenburg, Germany; 00 F-4424-E0) at 30°C column temperature. 20 mM KH_2_PO_4_ pH 2.5 (A) and 100% Methanol (B) were used as eluents to drive the following temporal gradients. 0–0.5 min 100% eluent A, 0.5-10 min increase of eluent B from 0% to 10%, 10–12 min decrease of eluent B from 10% back to 0% and 12–14 min again 100% eluent A. 10 μL sample was injected, a flow rate of 1 ml/min was adjusted and peaks were detected via UV at 220 nm.

### Lipid analysis

A 20 ml aliquot of the culture broth was centrifuged (4,700 rpm 5 min), the pellet was resuspended in saline (0.9% NaCl) and again centrifuged (4,700 rpm, 5 min). The supernatant was discarded and the pellet was freeze dried (−30°C, 0,370 mbar). Preparation for the quantitative and qualitative gas chromatographical analysis was done in a one-step-procedure by direct esterification plus extraction. A portion (20 mg) of freeze dried biomass was weighed into a 15 ml glass falcon with teflon cap. 1.5 ml hexane and 0.5 ml of 2 mg/ml internal standard (methyl benzoate) dissolved in hexane were added as solvent for the extraction of lipid. In addition, 2 ml 15% H_2_SO_4_ in methanol was added for the esterification step. Each sample was heated up to 100°C for 2 h with continuous shaking. After cooling on ice, 1 ml demineralised water was added. The mixture was centrifuged for 5 min at 2,500 rpm. 1 μl of the upper phase, containing the fatty acid methyl esters extract, was analyzed via chromatography (Agilent Technologies, 6890 N Network GC-System). The instrument was equipped with a DB-Wax column (l: 30 m d: 0.25 mm, Agilent Technologies Deutschland GmbH, Böblingen, Germany; 122–7032), a flame ionization detector and working with a pressure of 1.083 bar and initial temperature of 40°C. The column temperature was increased from 40°C to 250°C with a rate of 8°C/min. The temperature was held at 250°C for 10 min before cooling down to 40°C. The total fatty acid content and the identification of fatty acids were performed using the standard RM3 FAME Mix (Sigma Aldrich, Taufkirchen, Germany; 07256-1AMP) and Marine FAME Mix (Acid Methyl Ester Marine Oil FAME Mix) (Restek GmbH, Bad Homburg, Germany; 35066). Fatty acids which represented less than 1% of total fatty acids were combined to “trace fatty acids”.

### Accession numbers

The following EMBL accession numbers have been assigned for the fungal ribosomal genes of the isolates: CPOH4 *Cryptococcus podzolicus* as HG737350, SSOH12 *Pichia segobiensis* as HG737349 and TPST6 *Trichosporum porosum* as HG737348.

## Results

### Screening and identification of yeast isolates

Four different yeast strains in total could be isolated from three soil samples. Three isolates arose from peat bog samples and one isolate (TPST6) from grass land soil sample. Peat bog soil is assumed to contain a high level of carbon compared to nitrogen. Three out of those four yeast isolates were identified as potential lipid producers using the Sudan Black B staining technique on agar plates supplemented with antibiotic. The sequences of ITS region were compared with the nucleotide data base using the NCBI-blast tool. SSOH12 showed highest genetic agreement with *Pichia segobiensis* closely followed by *Scheffersomyces stipitis* which is a synonym of *Pichia stipitis*. CPOH4 was identified as *Cryptococcus podzolicus*. TPST6 was identified as *Trichosporon porosum*. The results are presented in Table 1. The fourth isolated yeast which could not be identified as oleaginous showed highest agreement to *Candida shehatae*, but was not further investigated in this study.

**Table 1:** Results of the sequencing of ITS region including 5.8 S rRNA of isolates SSOH12, CPOH4 and TPST6

**Isolate**	**Deposited at DSMZ as (EMBL accession number)**	**Total score**	**Query coverage [%]**	**E-value**	**Max identity**	**Closest relative in NCBI data bank (accession number)**
SSOH12	DSM 27193 (HG737349)	1062	99	0	99	*Pichia segobiensis* (DQ409166.1)
SSOH12	DSM 27193 (HG737349)	1059	99	0	99	*Scheffersomyces stipitis* (JQ026363.1)
CPOH4	DSM 27192 (HG737350)	852	100	0	100	*Cryptococcus podzolicus* (HF558652.1)
TPST6	DSM 27194 (HG737348)	878	100	0	100	*Trichosporon porosum* (HF558656.1)

*DSM*Z: German Collection of Microorganisms and Cell Cultures, EMBL: The European molecular Biology Laboratory, NCBI: National Center for Biotechnology Information.

### Shake flask cultivation

**Figure 1:**
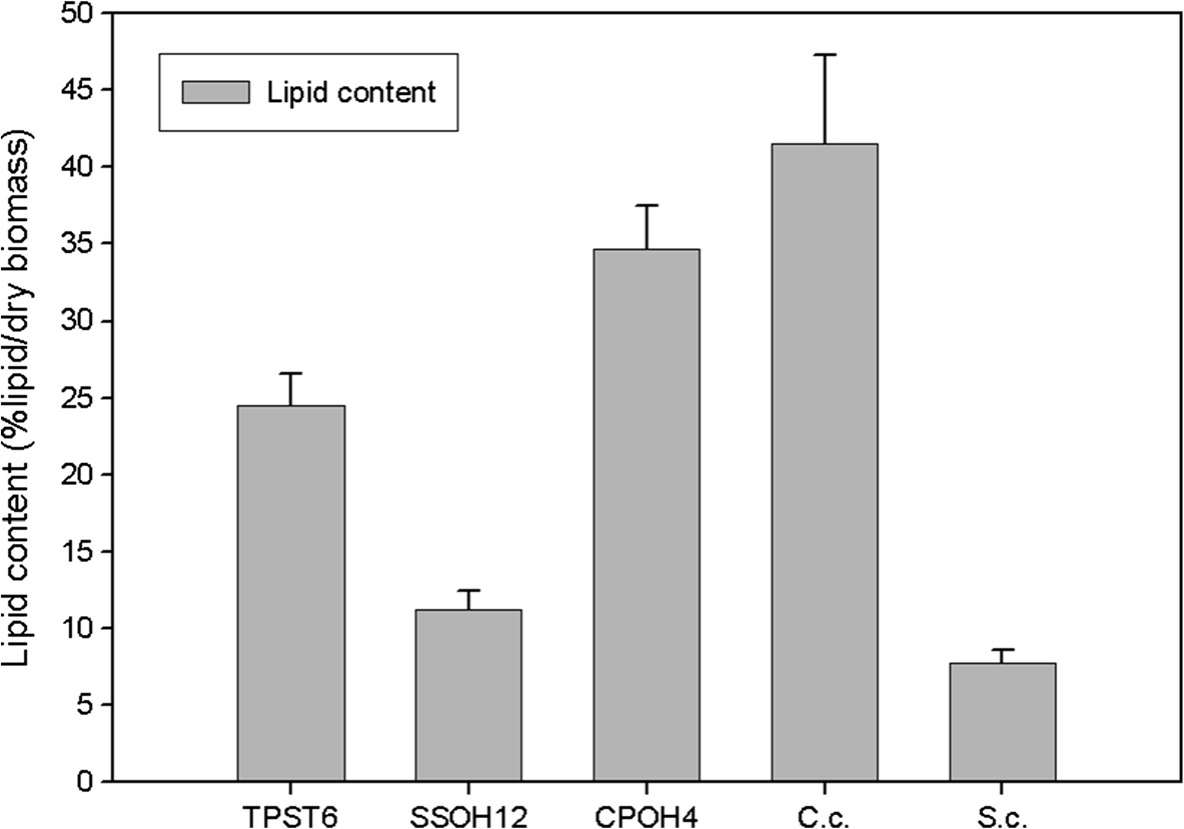
**Lipid content in shake flask cultivation at 25°C in YM medium after 120 h of all four isolates TPST6, CSOH1, SSOH12 and CPOH4 as well as oleaginous yeast** ***Cryptococcus curvatus*** **(*C.c.*) and non oleaginous yeast** ***Saccharomyces cerevisiae*** **(*S.c.*).**

All isolates were cultivated in 500 ml shake flasks containing YM medium supplemented with 50 g/L glucose. The flasks were incubated at 25°C for 120 hours. *C. curvatus,* a well known oleaginous yeast, was taken as the positive control and *S. cerevisiae* as the negative control. Glucose was taken as standard carbon source and was held in excess throughout the fermentation period. The pH value did not decrease below 4 in all cultivations. The results are presented in Figure 1. As expected, the oleaginous yeast *C. curvatus* yielded more than 40% lipid per dry biomass whereas *S. cerevisiae*, a typical non-oleaginous microorganism, yielded less than 10% lipid per dry biomass corresponding to the normal cellular amount of lipids.

The best lipid producer among the three new isolates was CPOH4 with 34.6% lipid per dry biomass and can therefore be classified as oleaginous under the chosen conditions in YM medium with additional glucose and shake flasks. TPST6 yielded 24.5% lipid per dry biomass, hence this yeast also belongs to the oleaginous microorganisms. However, SSOH12 yielded 11.2% lipid per dry biomass; hence SSOH12 cannot be classified as lipid producing strain under the chosen conditions in shake flasks with YM medium with additional glucose.

### Cultivation of the three isolated yeasts in 2.5 L-fermentor on glucose or xylose

**Figure 2:**
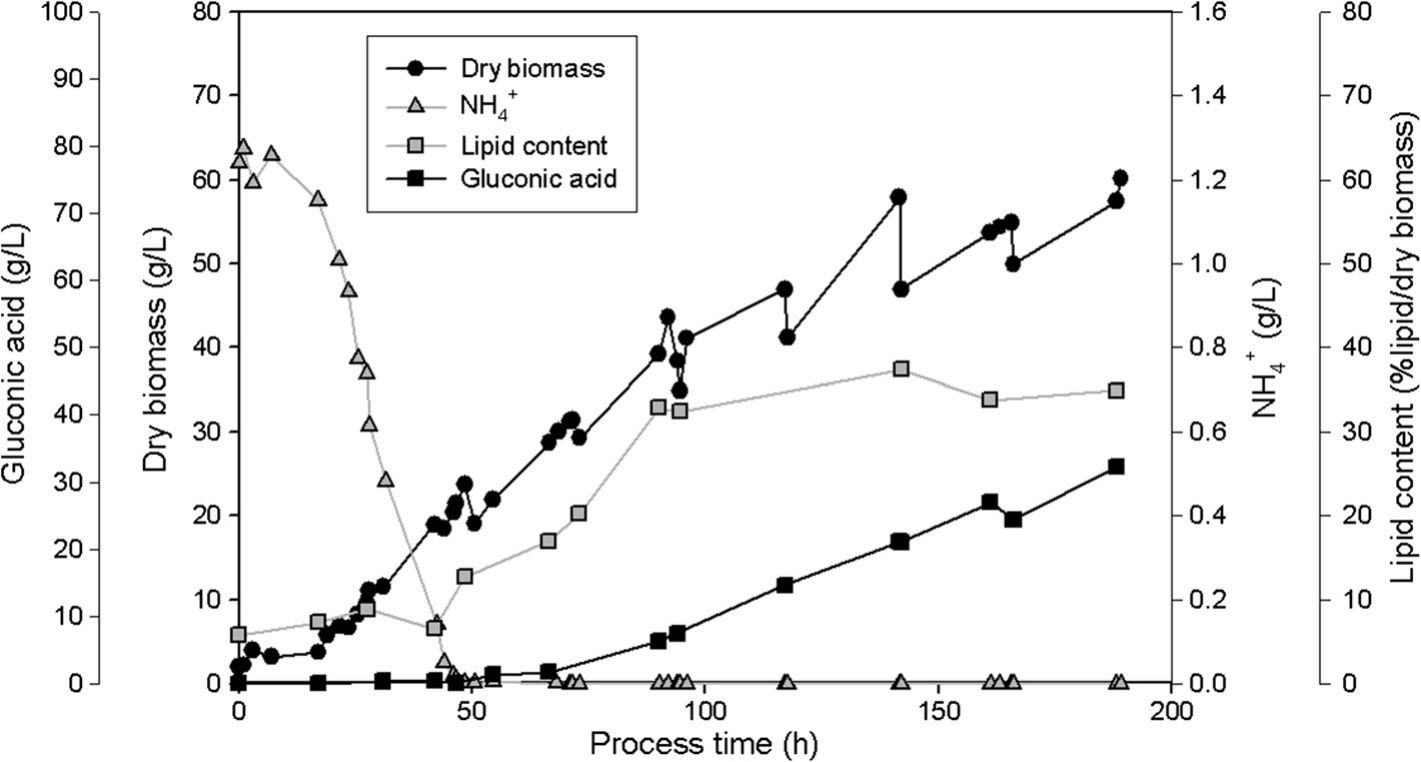
**CPOH4 cultivated on glucose in 2.5 L-bioreactor in mineral salt medium at pH 5 and 20°C; glucose was fed daily; NH**_**4**_^**+**^**consumption, production of biomass, lipid and gluconic acid are presented.**

**Figure 3:**
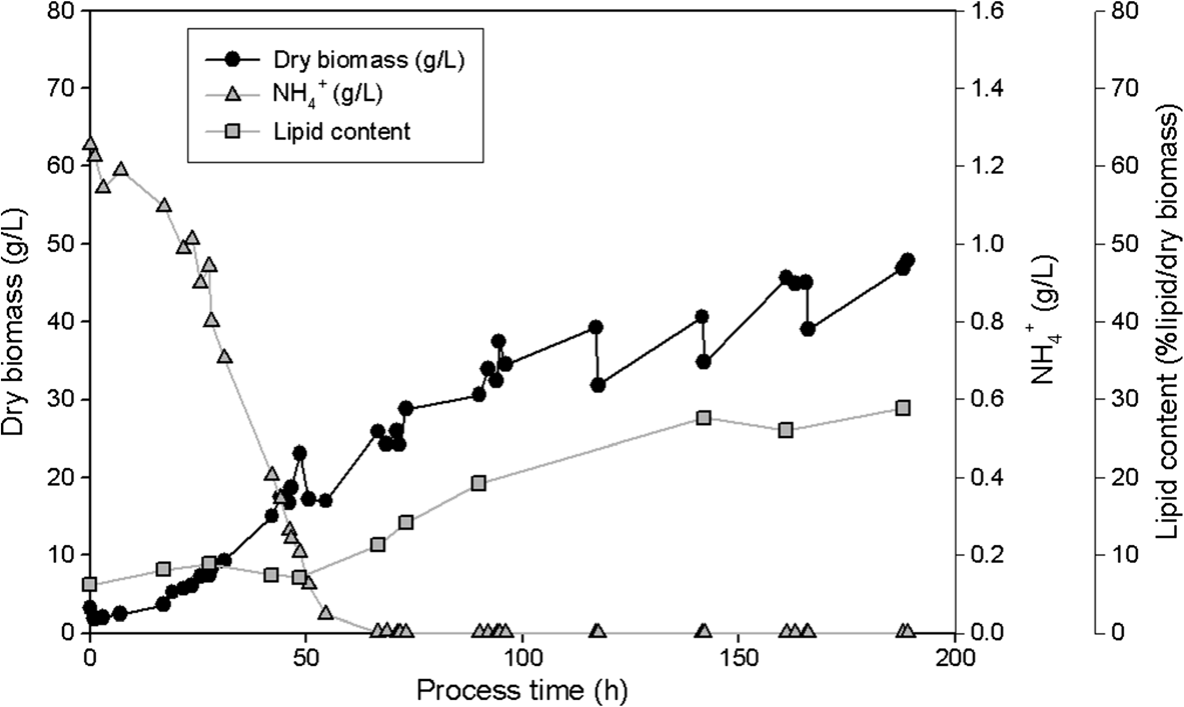
**CPOH4 cultivated on xylose in 2.5 L-bioreactor in mineral salt medium at pH 5 and 20°C; xylose was fed daily.** NH_4_^+^ consumption, production of biomass and lipid are presented.

**Figure 4:**
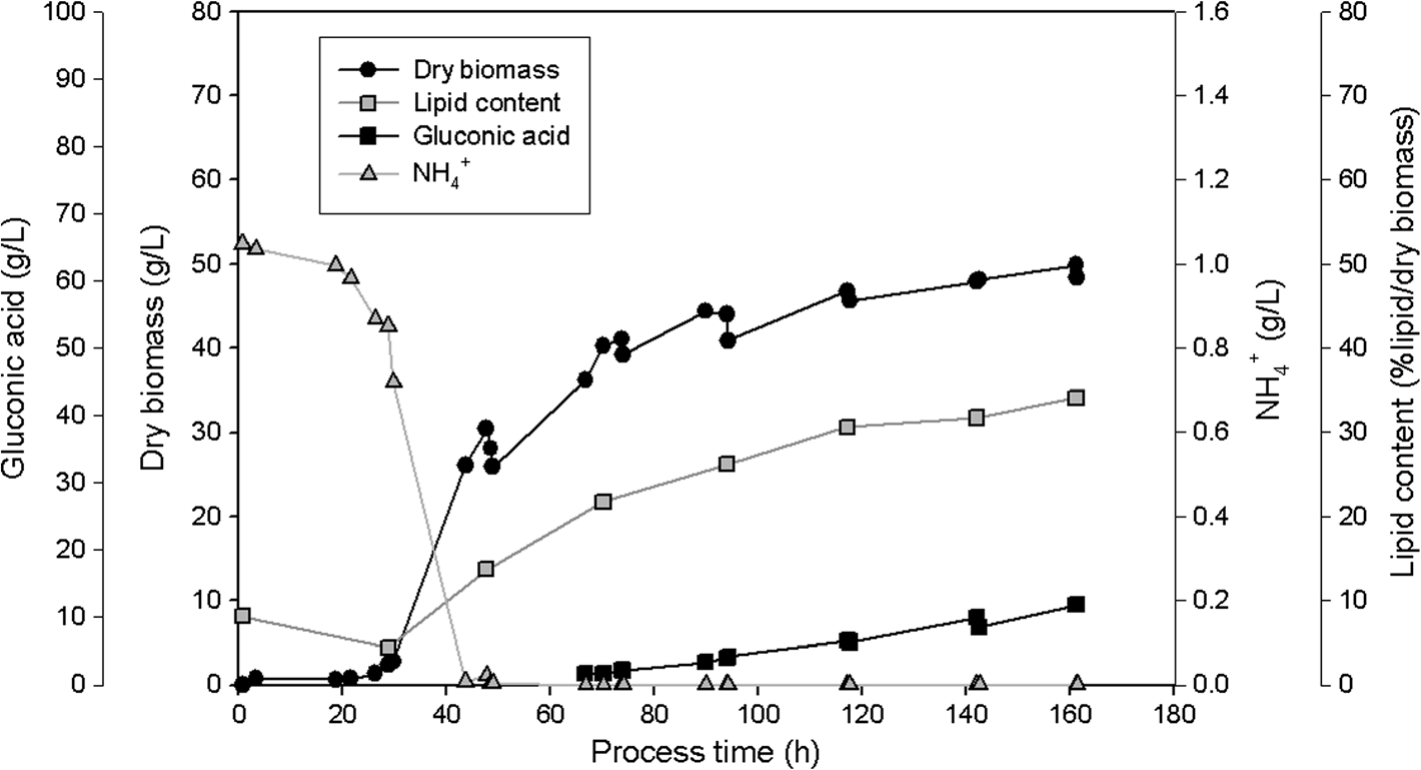
**TPST6 cultivated on glucose in 2.5 L-bioreactor in mineral salt medium at pH 5 and 25°C; glucose was fed daily.** NH_4_^+^ consumption, production of biomass, lipid and gluconic acid are presented.

**Figure 5:**
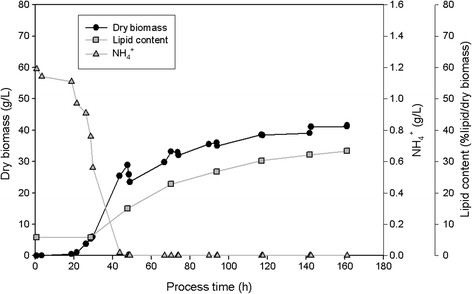
**TPST6 cultivated on xylose in 2.5 L-bioreactor in mineral salt medium at pH 5 and 25°C; xylose was fed daily.** NH_4_^+^ consumption, production of biomass and lipid are presented.

**Figure 6:**
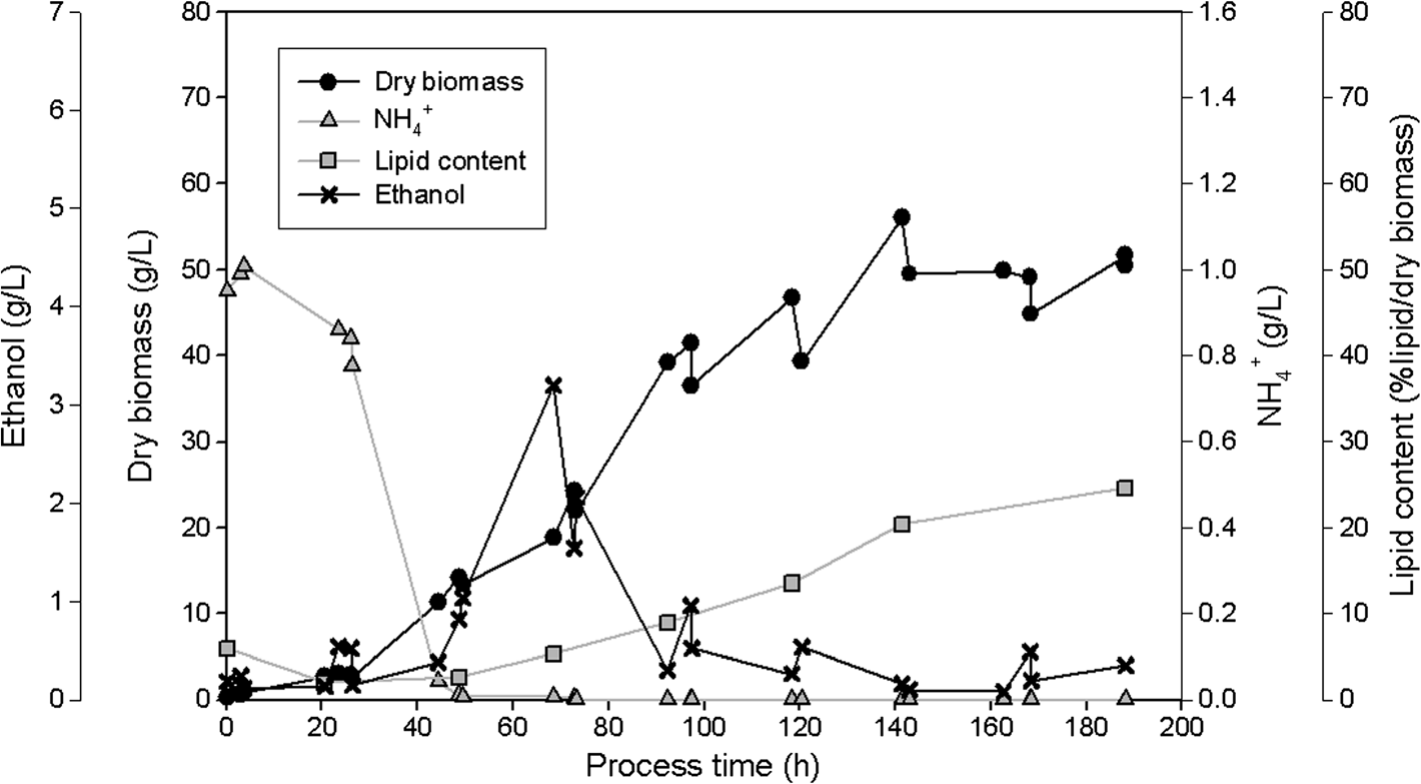
**SSOH12 cultivated on glucose in 2.5 L-bioreactor in mineral salt medium at pH 5 and 25°C; glucose was fed daily.** NH_4_^+^ consumption, production of biomass, lipid and ethanol are presented.

All three isolates SSOH12, CPOH4 and TPST6 were cultivated in bioreactors at pH 5 and 600 rpm during the whole cultivation. Hence, compared to shake flask cultivation a constant pH and higher aeration rates were ensured. CPOH4 was cultivated at 20°C in preliminary studies (data not shown) revealed best growth at this temperature. SSOH12 and TPST6 were cultivated at 25°C as this was the suggested cultivation temperature for *T. porosum* (Middelhoven et al. [2001]) and described growth conditions for *S. segobiensis* at ATCC data bank. The carbon source, glucose or xylose, was held in excess throughout the fermentation, but less than 90 g/L to prevent substrate inhibition. All strains were cultivated on glucose as the carbon source, but CPOH4 and TPST6 were also grown on xylose. Table 2 summarizes the main results of the different fermentations; growth yield coefficient (y_x/s_), product yield coefficient (y_p/s_), volumetric productivity (Q_L_), lipid content per dry biomass (%lipid/dry biomass), lipid concentration (g/L) and detected by-products are presented. The data for CPOH4 on glucose and xylose in Table 2 represent the mean from two independent fermentations. The data for TPST6 on glucose and xylose as well as the data for SSOH12 on glucose are single fermentations. To illustrate the lipid production processes in more detail the fermentations of all three isolates are exemplarily shown for one single fermentation in Figures 2, 3, 4, 5 and 6. Due to daily feeding of the carbon source in the case of CPOH4 and SSOH12 the values for the dry biomass are shown before and after feeding. Concerning TPST6, feeding of the carbon source was repeated only two times after 26 and 48 h. A third feeding was performed after 94 h only for the cultivation with glucose. For all isolates the lipid production started as soon as the nitrogen source NH_4_^+^ was totally consumed (approximately after 50 hours). The dissolved oxygen level reached minimum values of 0% during the maximal growth phase, but increased again as soon as the dry biomass started to stagnate. All three yeast isolates exceeded a lipid content of more than 20% lipid per dry biomass (Table 2). The highest lipid content was reached with yeast isolate TPST6. Using glucose as carbon source this strain produced up to 33.4% lipid per dry biomass, followed by CPOH4 (31.8%) and SSOH12 (24.6%). When cultured on xylose CPOH4 gave 26.8% lipid per dry biomass, whereas TPST6 yielded even 33.4%. Under the chosen conditions of the fermentations all three isolates may be classified as oleaginous yeasts. The determination of by-products was carried out as there were some indications for ethanol and acid production. In the culture broth of SSOH12 ethanol was detected whereas ethanol production in the case of CPOH4 and TPST6 was negligible (Table 2). Instead, CPOH4 and TPST6 showed evidence of acid production when cultured on glucose. This acid was identified as gluconic acid by HPLC analysis and was confirmed with an enzymatic test. Malic, citric, succinic and fumaric acids were not detected. These acids were neither detected for SSOH12 cultured on glucose nor for CPOH4 and TPST6 cultured on xylose. Cultured on glucose, CPOH4 yielded the highest lipid concentration with 18.0 g/L and the highest concentration of gluconic acid with 30 g/L, followed by TPST6 with a lipid concentration of 17.0 g/L and 12 g/L gluconic acid. SSOH12 resulted only in 12.7 g/L lipid and a minimum of 3.3 g/L ethanol as by-product. Considering the lipid productivity (Q_L_), TPST6 grown on glucose may be the best lipid producer (0.10 g/Lh) followed by TPST6 on xylose and CPOH4 on glucose with the same value of 0.09 g/Lh.

**Table 2:** Overview about performed fermentations of isolates SSOH12, CPOH4 and TPST6 at pH 5 in mineral salt medium; SSOH12 and TPST6 represent single fermentations, whereas CPOH4 are mean of two independent fermentations including standard deviations

**Isolate**	**SSOH12**	**CPOH4**		**TPST6**	
**Carbon source**	Glucose	Glucose	Xylose	Glucose	Xylose
**Process time [h]**	188	188		161	
**T [°C]**	25	20		25	
**Dry biomass**_**max**_**[g/L]**	51.7	56.5 ± 5.2	41.5 ± 1.24	49.9	41.5
**Y**_**x/s**_**[g/g]**	0.35	0.50 ± 0.0	0.53 ± 0.02	0.38	0.41
**Y**_**p/s**_**[g/g]**	0.06	0.11 ± 0.01	0.09 ± 0.0	0.11	0.12
**Q**_**L**_[g/L*h]	0.07	0.09 ± 0.04	0.07 ± 0.01	0.1	0.09
**Lipid content [%lipid/dry biomass]**	24.6	31.8 ± 8.0	26.8 ± 1.2	34.1	33.4
**Concentration of lipid [g/L]**	12.7	18	11.1	17	13.9
**Gluconic acid [g/L]**	n.d.	30	n.d.	12	n.d.
**Ethanol [g/L]**	>3.3	< 1.0	<1.0	< 1.0	<1.0

### Fatty acid profile

The analysis of the fatty acid profile (Table 3) revealed different profiles for each isolate. The main fatty acid in all isolates was oleic acid (C18:1) with content ranging between 39.6% and 59.4%. Relatively high yields of palmitic acid (C16:0) ranging from 18.4% to 21.1% were obtained in all three isolates. In addition to oleic and palmitic acid, palmitoleic acid (C16:1) with 16% was one of the main products in SSOH12 whereas CPOH4 and TPST6 produced only negligible amounts of this fatty acid. In contrast, TPST6 was characterized by high amounts of stearic acid (C18:0) and linolic acid (C18:2) ranging between 15.5% and 18.7%. CPOH4 produced 5% of stearic acid (C18:0) and just 8.7 to 11.1% of linolic (C18:2) acid. The amount of linolenic acid (C18:3) was less than 1.3% and is therefore negligible in all isolates.

**Table 3:** Fatty acid profile of the three isolates SSOH12, CPOH4 and TPST6 with respect to chosen carbon source employed in growth medium; amounts of fatty acids are given in% on total fatty acids; fatty acids detected with amounts less than 1% are combined to “Trace fatty acids”

**Isolate**	**Carbon source**	**Type of fatty acid (% of total fatty acids)**						
		**C16:0**	**C16:1**	**C18:0**	**C18:1**	**C18:2**	**C18:3**	**Trace fatty acids**
SSOH12	Glucose	19.1	16.0	2.0	51.8	7.5	0.5	3.1
CPOH4	Glucose	18.4	0.3	5.3	59.4	8.7	0.9	7.0
	Xylose	20.1	0.4	4.7	55.1	11.1	1.1	7.5
TPST6	Glucose	19.5	0.3	17.0	40.4	17.8	1.3	3.7
	Xylose	21.1	0.3	15.5	39.6	18.7	1.0	3.8

There was no remarkable difference in most of the fatty acid profile comparing glucose and xylose as the carbon source. However, for both isolates CPOH4 and TPST6 a slightly higher amount of oleic acid (C18:1) and slightly lower amount of linolic acid (C18:2) was observed when cultured on glucose.

## Discussion

Three new oleaginous yeast strains were isolated from soil samples arising from a peat bog and grassland using Sudan Black B staining on agar plates. The reason for peat bog soil was the fact that it contains high amount of carbon arising from old wood and plants while nitrogen content is low. The use of a grassland sample was chosen as a comparison sample without any specific limitations.

The first strain SSOH12 was identified as *Pichia segobiensis* producing a considerable amount of 16% palmitoleic acid (C16:1) of total lipid amount. The second strain CPOH4 was identified as *Cryptococcus podzolicus* and yielded the highest lipid concentration with 18.0 g/L of all isolates. The third strain TPST6 was identified as *Trichosporon porosum* and produced a considerable amount of 17.8% linolic acid (C18:2) on glucose and 18.7% on xylose. All three strains have not been described as oleaginous before. Due to sufficient production of lipid amounts and interesting fatty acid profiles, further studies of all three strains are worthwhile to establish sustainable bioprocesses for the production of adequate amounts of microbial oil for industrial applications.

### Influence of cultivation conditions on lipid production for screening experiments

Different lipid contents per dry biomass were reached depending on the manner of cultivation, if choosing a shake flask or a bioreactor*. C. podzolicus* CPOH4 and *T. porosum* TPST6 were able to produce high amounts of lipid between 24.5% and 34.1% lipid per dry biomass in both cultivation methods. In contrast, *P. segobiensis* SSOH12 with just 11.2% lipid per dry biomass could not be classified as oleaginous yeast when cultured in shake flasks with the chosen medium. However, when cultured in a 2.5 L-bioreactor 24.6% lipid per dry biomass was realized with *P. segobiensis* SSOH12, which corresponds to twice the amount obtained with shake flask cultivation. Low aeration rates in shake flasks may be the reason for low lipid level since increasing cell densities lead to less available oxygen. Moreover, a nitrogen limiting mineral salt medium with adequate buffering capacities would be more appropriate for shake flask cultures. Under low aeration rates facultative anaerobic strains start to produce ethanol. It is thought that the oxygen limitation during the maximal growth phase in the cultivations with bioreactors occurred as the agitation speed was too low for the present cell density. In the present study *P. segobiensis* SSOH12 produced ethanol as by-product as soon as the dissolved oxygen level reached 0% (data not shown). Hence, this strain may be classified as a facultative anaerobic yeast. This shows the importance of adequate aeration even for screening experiments. To prevent the production of ethanol and to increase the production of lipid by higher dissolved oxygen the agitation speed has to be increased. Further experiments should be done with controlled dissolved oxygen. Strains that gave a positive test result with Sudan Black B staining on agar plates, but a negative result in shake flasks with YM medium supplemented with glucose, are worthwhile to examine in further detail in a well aerated bioreactor as not only the nitrogen limitation but also the sufficient oxygen supply may be a prerequisite for lipid production.

In addition to the oxygenation rate, the pH of the medium is also an important parameter to consider during the screening of lipid producing strains as the pH value cannot be controlled in shake flasks. Most strains are acid labile and would not grow well in acidic environment like *Candida shehatae* (Kastner et al. [1996]), and therefore would even not produce any favored lipid. The shake flask cultivation of *C. curvatus* and *C. podzolicus* CPOH4 in mineral salt medium (data not shown) resulted in a decrease of the pH value from 5.0 to 2.0 within 60 hours. The well known oleaginous yeast *C. curvatus* produced just a maximum of 15% lipid per dry biomass under these conditions whereas *C. podzolicus* CPOH4 ended up with 23% lipid within 100 h cultivation time (data not shown). The advantage of *C. podzolicus* CPOH4 is its acid resistance, which is beneficial in podsol soil, an acidic environment, from which *C. podzoilicus* CPOH4 could be isolated and from which its name arose (Botes et al. [2005]). Cultivating *C. curvatus* in YM medium the pH decreased only from 5 to 4 and yielded up to more than 40% lipid per dry biomass. This shows that the YM medium supplemented with glucose has good buffering conditions and is a useful medium for a first examination of yeast strains.

### Characterization of newly isolated strains

SSOH12 was identified as *Pichia segobiensis*. This strain belongs to the ascomycetes. The second highest agreement of SSOH12 was with *Scheffersomyces stipitis,* also known as *Pichia stipitis. Pichia stipitis* (Nigam [2001]) and *Scheffersomyces stipitis* (Liu et al. [2010]) are well described within the context of microbial ethanol production, but have not been mentioned previously with regards to lipid production. Furthermore they are able to assimilate hemicellulosic compounds (Ferreira et al. [2011]; Nigam [2001]).

Aside from the focus on the production of ethanol under anaerobic conditions, this study has shown that *P. segobiensis* SSOH12 is able to produce more than 24% lipid content under aerobic conditions and sufficient oxygenation. As the ethanol production of *P. segobiensis* SSOH12 commences only after oxygen limitation occurs, the crabtree effect like in *Saccharomyces cerevisiae* (Al-mhanna [2010]) is excluded. This could be confirmed for the closely related yeast, *Scheffersomyces stipitis* (Papini et al. [2012]). Under aerobic conditions this yeast can be classified as oleaginous yeast and process optimization with higher dissolved oxygen level might increase the lipid yield.

The special feature of *P. segobiensis* SSOH12 concerning the fatty acid profile is the different composition to most other oleaginous yeast containing a fatty acid similar to cacao-butter with the main components of C16:0, C18:0, C18:1 and C18:2. In addition, *P. segobiensis* SSOH12 comprises 16% palmitoleic acid (C16:1), an omega-7 mono-unsaturated fatty acid which has been shown to have positive effects against obesity (Yang et al. [2011]) and potential for the prevention of brain and cardiovascular diseases (Matsunaga et al. [1995]). It is a component of some oil seeds, especially sea-buckthorn (Fatima et al. [2012]) or macadamia (Nestel et al. [1994]). An alternative source via microbial production is the opportunity to produce palmitoleic acid in sufficient quantities for possible future applications in medicine. *P. segobiensis* SSOH12 has been described as one of the best xylose-converting strains (Liu et al. [2010]; Toivola et al. [1984]). Hence, the investigation of lipid production with xylose as the carbon source may be a worthwhile exercise.

The two other isolated strains were identified as *Cryptococcus podzolicus* (CPOH4) and *Trichosporon porosum* (TPST6). Both strains belong to the yeast-like anamorphic basidiomycetes and are found in soil (Botes et al. [2005]; Colombo et al. [2011]). They are also known to assimilate hemicelluloses (Middelhoven et al. [2001]; Shubakov [2000]).

*Trichosporon* sp. and *Cryptococcus* sp. in general are known to belong to the oleaginous strains (Gujjari et al. [2011]; Zhu et al. [2008]; Hu et al. [2011]), whereas *T. porosum* and *C. podzolicus* have not been mentioned before in relation to microbial oil production. The results of this study reveal that both strains are able to produce at least 30% lipid per dry biomass when grown on glucose or xylose as carbon source. Therefore, *C. podzolicus* CPOH4 and *T. porosum* TPST6 can be characterized for the first time as oleaginous yeasts. *T. porosum* TPST6 produced in this study almost 20% linolic acid which makes it unique among other *Trichosporon* species which generally yield less than 10%, e.g. *Trichosporon fermentans* less than 8% (Huang et al. [2012]) and *Trichosporon cutaneum* less than 3.4% (Hu et al. [2011]).

The first assumption to explain the observed acid production in the culture broth of CPOH4 and TPST6 was that excessive citric acid may be secreted into the medium which serves as a precursor for Acetyl-CoA and further for the production of triacylglycerides in oleaginous strains (Ratledge [2002]). However, no citric acid could be determined in this study. Instead, gluconic acid was measured as additional by-product with high concentrations up to 30 g/L for *C. podzolicus* CPOH4 and 12 g/L for *T. porosum* TPST6. Both strains are simultaneous producers of lipid and gluconic acid.

Gluconic acid and its derivates find wide application in the food and pharmaceutical industries. Therefore it could be worthwhile to improve gluconic acid production with the newly isolated yeasts *C. podzolicus* CPOH4 or *T. porosum* TPST6. The ascomycete *Aspergillus niger* (Ramachandran et al. [2006]) and yeast like *Aureobasidium pullulans* (Anastassiadis and Rehm [2006]) are other examples of gluconic acid producers with high production rates of 120–140 g/L and up to 370 g/L, respectively.

*C. podzolicus* CPOH4 and *T. porosum* TPST6 give the opportunity to favor either the production of intracellular oil or the production of gluconic acid or rather the simultaneous production. An advantage of the simultaneous production of lipid and gluconic acid would be the easy separation of both products as the oil is produced intracellular, whereas the gluconic acid is secreted into the culture broth. Higher aeration rates to prevent oxygen limitation are required in any case to increase the product levels, although ethanol production is negligible. Negligible ethanol production which started just at the stage of oxygen limitation verifies that both strains are crabtree negative. To avoid the production of gluconic acid as by-product in a lipid production process, xylose may be the carbon source of choice. However, another interesting approach could be the combined feeding of glucose and xylose as both carbon sources are components of hydrolyzed wood and straw waste. If glucose and xylose were consumed simultaneously hydrolyzed straw and wood wastes could be used as low-cost carbon source. *Trichosporon cutaneum* (Hu et al. [2011]) and *Cryptococcus curvata* (Heredia and Ratledge [1988]) could be described as such strains. The use of other low-cost carbon sources and further process optimization to increase the lipid yield are further possible approaches.

Both isolates *C. podzolicus* CPOH4 and *T. porosum* TPST6 are suitable for a lipid production bioprocess, but *T. porosum* TPST6 shows a more interesting fatty acid profile with 18.7% linoleic acid (C18:2). Moreover, xylose as carbon source favours the lipid production whereas glucose as carbon source leads to a simultaneous production of gluconic acid and intracellular lipid.

*P. segobiensis* is worthwhile to be further investigated because of its considerable amount (16%) of plamitoleic acid (C16:1) which may be suitable for medical applications.

## Competing interests

The authors declare that they have no competing interests.
